# Pretreatment with mGluR2 or mGluR3 Agonists Reduces Apoptosis Induced by Hypoxia-Ischemia in Neonatal Rat Brains

**DOI:** 10.1155/2021/8848015

**Published:** 2021-03-06

**Authors:** Ewelina Bratek - Gerej, Agnieszka Bronisz, Apolonia Ziembowicz, Elzbieta Salinska

**Affiliations:** ^1^Department of Neurochemistry, Mossakowski Medical Research Institute Polish Academy of Sciences, Warsaw, Poland; ^2^Tumor Microenvironment Laboratory, Mossakowski Medical Research Institute Polish Academy of Sciences, Warsaw, Poland

## Abstract

Hypoxia-ischemia (HI) in an immature brain results in energy depletion and excessive glutamate release resulting in excitotoxicity and oxidative stress. An increase in reactive oxygen species (ROS) production induces apoptotic processes resulting in neuronal death. Activation of group II mGluR was shown to prevent neuronal damage after HI. The application of agonists of mGluR3 (N-acetylaspartylglutamate; NAAG) or mGluR2 (LY379268) inhibits the release of glutamate and reduces neurodegeneration in a neonatal rat model of HI, although the exact mechanism is not fully recognized. In the present study, the effects of NAAG (5 mg/kg) and LY379268 (5 mg/kg) application (24 h or 1 h before experimental birth asphyxia) on apoptotic processes as the potential mechanism of neuroprotection in 7-day-old rats were investigated. Intraperitoneal application of NAAG or LY379268 at either time point before HI significantly reduced the number of TUNEL-positive cells in the CA1 region of the ischemic brain hemisphere. Both agonists reduced expression of the proapoptotic Bax protein and increased expression of Bcl-2. Decreases in HI-induced caspase-9 and caspase-3 activity were also observed. Application of NAAG or LY379268 24 h or 1 h before HI reduced HIF-1*α* formation likely by reducing ROS levels. It was shown that LY379268 concentration remains at a level that is required for activation of mGluR2 for up to 24 h; however, NAAG is quickly metabolized by glutamate carboxypeptidase II (GCPII) into glutamate and N-acetyl-aspartate. The observed effect of LY379268 application 24 h or 1 h before HI is connected with direct activation of mGluR2 and inhibition of glutamate release. Based on the data presented in this study and on our previous findings, we conclude that the neuroprotective effect of NAAG applied 1 h before HI is most likely the result of a combination of mGluR3 and NMDA receptor activation, whereas the beneficial effects of NAAG pretreatment 24 h before HI can be explained by the activation of NMDA receptors and induction of the antioxidative/antiapoptotic defense system triggered by mild excitotoxicity in neurons. This response to NAAG pretreatment is consistent with the commonly accepted mechanism of preconditioning.

## 1. Introduction

Hypoxic-ischemic encephalopathy (HIE) is one of the major causes of human infant mortality and morbidity, contributing to neuronal injury and impaired development [1], affecting up to 1.2 million infants annually [2].

Only a minority of infants with severe encephalopathy after perinatal asphyxia survive without handicap. A large number of infants who do survive neonatal hypoxia-ischemia (HI) exhibit cerebral palsy, developmental delays, visual and hearing impairment, and learning and behavioral problems [3]. There are very few protective treatments, and the only licensed treatment that is currently used is hypothermia. However, hypothermia used alone appears to be insufficient to prevent brain injury; therefore, understanding the molecular mechanisms contributing to neuropathology resulting from HI is so important.

HI in the immature brain causes a depletion of energy sources that results in neuron and glial cell depolarization and the release of glutamate. This leads to excessive N-methyl-D-aspartate receptor (NMDA) activation that initiates an intracellular cascade of events leading to neuronal death, including the development of oxidative stress and apoptotic processes [4]. Oxidative stress results from hypoxia/ischemia-induced disturbances in mitochondrial functioning that lead to increased production of reactive oxygen species (ROS). It was shown that increased ROS production triggers apoptotic signals that include the Bcl-2 family proapoptotic Bax protein and activation of caspases [5].

Hypoxic conditions increase the intracellular level of hypoxia-inducible factor 1 alpha (HIF-1*α*), an important regulator of hypoxia-induced apoptosis. HIF-1*α* acts in combination with many other factors, and it can be both pro- and antiapoptotic [6]. Under hypoxic/ischemic conditions, HIF-1*α* initiates apoptosis by increasing the expression of proapoptotic Bcl-2 family proteins, inhibiting the antiapoptotic effects of Bcl-2 [6]. It was shown that elevated ROS levels are involved in HIF-1*α* accumulation during hypoxia and that ROS/HIF-1*α* pathway participation in cell damage is not restricted only to hypoxia-ischemia [7–12].

Recent evidence suggests that metabotropic glutamate receptors (mGluRs), which are G-protein coupled receptors, may provide an effective alternative approach to reducing glutamate-mediated cell death [13, 14]. mGluRs regulate a variety of intracellular signaling systems, acting on both pre- and postsynaptic membranes [15, 16]. Group II mGluRs are presynaptic receptors on neurons (mGluR2/3), but they are also expressed on astrocytes and microglia (mGluR3) [17]. A key function of presynaptic mGluR2/3 is to reduce the release of neurotransmitters [18, 19]. Both receptor types are known to play a role in the modulation of synaptic plasticity, particularly in stimulating LTD (long-term depression) of excitatory synaptic transmission [20–22]. Recently, their potential involvement in neuroprotective processes after HI was postulated. In vivo experiments have shown that a highly selective group II agonist, LY379268, applied a short time after ischemic insult, was neuroprotective in a model of global ischemia in gerbils and a rat experimental model of birth asphyxia [13, 23, 24], suggesting a potential therapeutic role for mGluR2/3 activation.

N-Acetylaspartylglutamate (NAAG) is a highly selective endogenous agonist of mGluR3 [25] and a mixed agonist/antagonist of the NMDA receptor [26]. In an animal model of neonatal HI, systemic injection of NAAG up to 1 h after HI insult significantly reduced brain damage preventing neuronal death in the CA1 region of the hippocampus and decreasing HI-induced elevation in cAMP concentration [27]. In our previous study, we showed that application of NAAG or LY379268 a short time after HI reduced oxidative stress [13]. We also demonstrated that pretreatment of animals with each agonist up to 24 h before HI resulted in neuroprotection and reduced ROS production, although the molecular mechanisms of their activity seemed to be distinct [13, 28, 29].

The neuroprotective effect of mGluR2/3 activation may be the result of a presynaptic reduction in glutamate release as well as activation of mGluR3 receptors present on astrocytes and the production of neuroprotective factors [30]. Activation of mGluR2/3 induces increased expression of brain-derived neurotrophic factor (BDNF) in neurons and microglia [31, 32] and has been implicated in the production of another neurotrophic factor, TGF*β*1 [33, 34].

Recently, the specific protective pathways by which glial cells, particularly astrocytes, can protect the brain after ischemic insults have been identified. Their important role in the development of the phenomenon of “ischemic tolerance” was also strongly implicated [35]. In our recent paper, we suggested that the neuroprotective effect of NAAG pretreatment may be part of the induction of ischemic tolerance by activation of NMDA receptors ([29] in press).

The aim of this study was to investigate the potential antiapoptotic effects of group II mGluR activation prior to experimental HI in 7-day-old rats. The effect of the application of each agonist on HI-induced changes in the expression and activity of the main apoptotic factors was analyzed.

## 2. Materials and Methods

### 2.1. Ethics Approval and Consent for Participation

All experiments described in this study were approved by the 2nd Local Ethical Committee based in Warsaw, Poland, and were performed following Polish governmental regulations (Dz.U.2015. poz.266) and the European Community Council Directive 2010/63/EU. Each experiment was performed on 3 different litters (10-12 rats per litter), and animals were randomly selected for experimental groups (2-3 animals from each litter). All surgeries were performed under isoflurane anaesthesia, and all efforts were made to minimize animal suffering and the number of animals used (total number of animals used: 150). The mortality rate did not exceed 5%.

### 2.2. Experimental Hypoxia-Ischemia

Seven-day-old Wistar rat pups of both sexes were anaesthetized, and the left common carotid artery was exposed, double-ligated with silk sutures, and cut between the ligatures. After completion of the surgical procedure, animals were returned to their dams and allowed to recover for 1 h. Afterward, animals were placed in a chamber ventilated with the warm humidified hypoxic gas mixture (7.5% oxygen in nitrogen, 35°C) for 75 min. After completion of hypoxia exposure, pups were returned to their dams and housed at 20°C and 12/12 h light/dark cycle with food and water access ad libitum.

Control pups were sham-operated (i.e., anaesthetized and their left common carotid artery exposed but not ligated), placed in the hypoxic chamber, and ventilated with humidified air only.

### 2.3. Drug Application

mGluR2 agonist LY379268 (5 mg/kg) or the specific mGluR3 agonist NAAG (N-acetylaspartylglutamate; 5 mg/kg) was administered intraperitoneally (i.p.) 24 h or 1 h before hypoxia-ischemia (HI). The dose of agonists was determined based on previously published findings [27, 28]. Sham-operated and HI control rats were injected with saline.

### 2.4. Evaluation of Brain Damage

Brain samples from each experimental group were analyzed for apoptosis using TUNEL staining. Seven days after HI, animals were anaesthetized and intracardiac perfusion with 4% paraformaldehyde solution with phosphate-buffered saline (PBS) was performed (3–5 animals from each experimental group). Brains were removed and immersed in 4% formalin for 1 week, then transferred to absolute ethanol and embedded in paraffin. Next, brains were cut into 10 *μ*m cross-sections using a microtome. Selected sections were incubated with proteinase K (20 *μ*g/ml in 10 mM Tris-HCl (pH 7.5)) for 15 min at 37°C. The TUNEL reaction mixture containing DNA polymerase, terminal deoxynucleotidyl transferase (TdT), and labelled nucleotides (TUNEL, In Situ Cell Death Detection Kit, Fluorescein; Roche, Switzerland) was applied to the sections in a humidified chamber. Sections were incubated for 60 min at 37°C, washed in PBS, mounted in the “antifade” medium, and analyzed under a fluorescence microscope. Labelled neurons in the CA1 region of the hippocampus were counted in region 500 *μ*m in length using AxioVision visualization software (Carl Zeiss, Aalen, Germany). At least 6 sections from each animal (3 sections per slide) were analyzed.

### 2.5. Western Blot Analysis of Pro- and Antiapoptotic Protein Expression

Expression of the antiapoptotic protein Bcl-2 and proapoptotic Bax was determined using the western blot method.

Tissues from both hemispheres were collected 2 h after HI and homogenized separately in a PBS buffer containing 10 mM EGTA, 10 mM EDTA, 0.1 mM PMSF, 100 mM NaCl, and a mixture of protease inhibitors. Protein concentration was determined using the Bradford assay. Prepared samples (50 *μ*g of protein per 25 *μ*l of homogenate) underwent SDS-polyacrylamide gel electrophoresis, and the separated proteins were transferred to the nitrocellulose membranes. Membranes were incubated in 5% skimmed milk in TBS+0.05% Tween 20 for 60 minutes, washed, and then incubated overnight at room temperature with selected primary antibodies specific for the analyzed proteins (Bcl-2, rabbit polyclonal antibody, Cell Signaling; Bax, rabbit polyclonal antibody, Cell Signaling; *β*-actin, goat polyclonal antibodies, Abcam) at a dilution of 1 : 100 and 1 : 500 for *β*-actin. After rinsing, membranes were incubated for 1 h with appropriate secondary antibodies conjugated with alkaline phosphatase (Sigma-Aldrich). Protein bands were visualized using the Vector Blue Alkaline Phosphatase Substrate Kit (Vector Laboratories, USA), scanned using an Image Scanner III (GE Healthcare), and measured by densitometry using ImageQuant. Changes in protein expression are presented as a percentage of the control. *β*-Actin (Abcam, diluted 1 : 500) was used as an internal standard.

### 2.6. Determination of the Expression of Selected Proteins by ELISA

Tissues from the right and left hemispheres were collected 2 h after HI and homogenized separately in 50 mM potassium orthophosphate at pH 7.0 containing 1 mM EDTA. Homogenates were incubated in RIPA buffer for 1 h at 4°C; then, the samples were centrifuged for 10 min at 10 000 × g at 4°C. After centrifugation, the lysates were transferred to new cooled 1.5 ml polyethylene tubes. Protein concentration was measured (a Bradford method), and samples were frozen at -80°C for further determination.

Expression of hypoxia-inducible factor 1-alpha (HIF-alpha), as well as caspase-3 and caspase-9 activity, was determined using ELISA kits (Caspase-9 Assay Kit and Caspase-3 Assay Kit—Fluorometric, Abcam, HIF-1*α* ELISA Kit MyBioSource, USA) according to the manufacturer's instructions.

### 2.7. Statistical Analysis

Results are expressed as the mean values ± SEM of each experimental group. Statistical analysis was performed using one-way ANOVA with Dunnett's post hoc test for significant differences between groups (GraphPad Prism 5). Differences were considered statistically significant when the *p* value was less than 0.05.

## 3. Results

### 3.1. Pretreatment with mGluR2/3 Agonists Prevents Hippocampal Neuronal Damage

**Figure 1:**
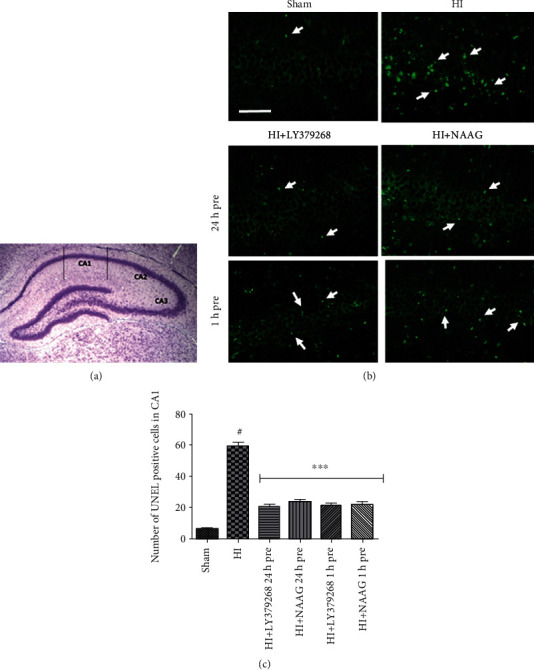
TUNEL-positive cells in the CA1 region of the ipsilateral hemisphere in mGluR2/3 agonist-preconditioned animals. Brain tissue was examined 7 days after ischemia. (a) TUNEL-stained cells were calculated from the central CA1 area of 0.5 mm length; (b) representative pictures of TUNEL-stained cells, scale bar: 50 *μ*m; (c) results expressed as the number of TUNEL-positive cells. The number of analyzed animals per group *n* = 3-5. The results on the graph are presented as the mean values ± SEM, statistically significant differences: ^∗∗∗^*p* < 0.001 compared to HI; ^#^*p* < 0.01 compared to the sham-operated group.

The number of TUNEL-positive cells was significantly increased in the CA1 region of the hippocampus in the ipsilateral (left) hemisphere after HI (60 ± 6 TUNEL-positive cells in the observed area) compared to the control group (5 ± 2 TUNEL-positive cells, *p* < 0.001, *F*_1,8_ = 204.98) (Figure 1).

LY379268 reduced the number of TUNEL-positive cells when applied 24 h or 1 h before HI (19 ± 3 and 22 ± 2, respectively, *p* < 0.001 compared to the HI group), and similar results were also observed after injection of NAAG (22 ± 1 and 20 ± 3 for pretreatment 1 h and 24 h before HI, respectively, *p* < 0.001).

mGluR2/3 agonists applied to sham-operated animals did not result in neuronal damage or an increase in the number of TUNEL-positive cells (data not shown).

### 3.2. The Effect of Pretreatment with mGluR2/3 Agonists on the Expression of Anti- and Proapoptotic Factors in response to HI

**Figure 2:**
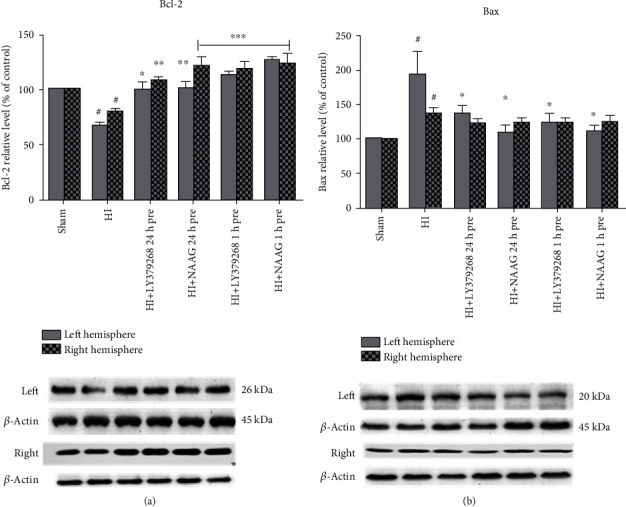
The effect of mGluR2/3 agonist application on HI induced changes in Bcl-2 (a) and Bax (b) expression in the brain. Results are presented as the mean ± SEM, *n* = 6–8; statistically significant differences: ^∗^*p* < 0.05, ^∗∗^*p* < 0.01, and ^∗∗∗^*p* < 0.001 compared to HI; ^#^*p* < 0.01 compared to the sham-operated group.

Levels of pro- and antiapoptotic factors after HI were markedly changed in both the ipsilateral and contralateral hemispheres as an effect of 75 min of hypoxia. We observed an HI-induced decrease in Bcl-2 expression in both hemispheres to 67.8% and 80% of the control in the left and right hemispheres, respectively (*p* < 0.001, *F*_1,10_ = 109.63 and *F*_1,10_ = 47.36, respectively) (Figure 2(a)) and a significant increase in Bax levels to 193% and 136% of the control in the left and right hemispheres, respectively (*p* < 0.01 for both groups) (Figure 2(b)). Application of both examined group II mGluR agonists not only inhibited the decrease in Bcl-2 but also evoked a significant increase in both hemispheres. LY379268 applied 1 h before HI increased Bcl-2 expression in both hemispheres to 113% and 119% of the control for the left and right hemispheres, respectively (*p* < 0.001 for both groups compared to the HI group). LY379268 application 24 h before HI inhibited the decrease in Bcl-2 in both hemispheres (100% and 109% of the control in the left and right hemispheres, respectively, *p* < 0.05 compared to the HI group).

Treatment of animals with NAAG 24 h before HI also resulted in inhibition of the decrease in Bcl-2 concentration that remained at the same level as a control in the left hemisphere (101%, *p* < 0.01 compared to HI) but increased significantly in the right hemisphere compared to the HI group (122%, *p* < 0.001); however, this Bcl-2 level was not significantly different from control values. Application of NAAG 1 h before HI resulted in a significant increase in Bcl-2 levels to 127% of the control in the left hemisphere (*p* < 0.001 compared to the control and HI groups) and 123% in the right hemisphere (*p* < 0.001 compared to the control group).

Application of LY379268 24 h before HI decreased Bax levels in both hemispheres to 132% and 123% of the control, respectively (*p* < 0.05 compared to the HI and sham groups). Application of LY379268 1 h before HI decreased Bax levels to 123% of the control in both hemispheres (*p* < 0.05 compared to the HI group). NAAG applied 24 h or 1 h before HI reduced Bax levels to values that were not significantly different from those of the sham group (110% and ~124% for the right and left hemispheres, respectively) but different from those of the HI group (*p* < 0.05 for the left and right hemispheres).

### 3.3. The Effect of Pretreatment with mGluR2/3 Agonists on Caspase-3 and Caspase-9 Activity after HI

**Figure 3:**
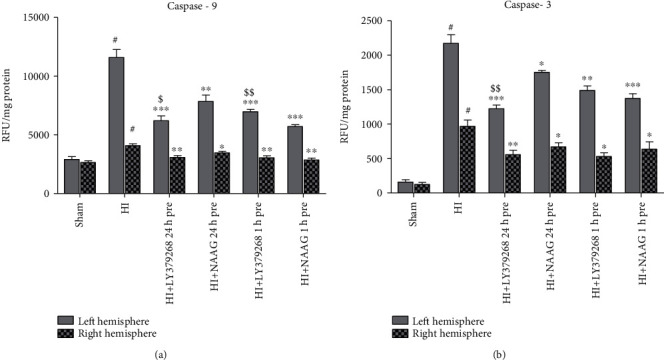
The effect of LY379268 or NAAG application 24 h or 1 h before HI on caspase-9 (a) and caspase-3 (b) activity. Results are presented as the mean ± SEM, *n* = 6–8; statistically significant differences: ^#^*p* < 0.001 compared to the sham-operated group. ^∗^*p* < 0.05, ^∗∗^*p* < 0.005, and ^∗∗∗^*p* < 0.001 compared to HI; ^$^*p* < 0.05 and ^$$^*p* < 0.001 compared to NAAG group.

HI significantly increased caspase activity in both hemispheres. Caspase-9 activity after HI increased to 400% and 153% of the activity observed in the sham group in the left and right hemispheres, respectively (Figure 3(a)), and the activity of caspase-3 increased to 1380% and 958% of the control in the left and right hemispheres, respectively (Figure 3(b)). Pretreatment with each of the agonists reduced the activities of caspase-3 and caspase-9 after HI insult. Application of LY379268 24 h before HI reduced caspase-9 and caspase-3 activity by 46% and 43%, respectively, and this reduction was significantly higher than that observed after NAAG application (32% and 19% for caspase-9 and caspase-3, respectively; *p* < 0.05 and *p* < 0.001, respectively).

LY367268 applied 1 h before HI significantly reduced the activity of caspase-9 in the left hemisphere by 36%, whereas NAAG application at the same time reduced the activity of this enzyme by 50%, and the effect of NAAG was significantly stronger than that of LY379268 (*p* < 0.001, *F*_1,8_ = 27.64).

Activity of caspase-9 in the right hemisphere was significantly decreased by both agonists and remained at the control level, while caspase-3 activity remained on average level 5-fold higher than the control.

### 3.4. Effect of Pretreatment with mGluR2/3 Agonists on HIF-Alpha Level after Hypoxia-Ischemia

**Figure 4:**
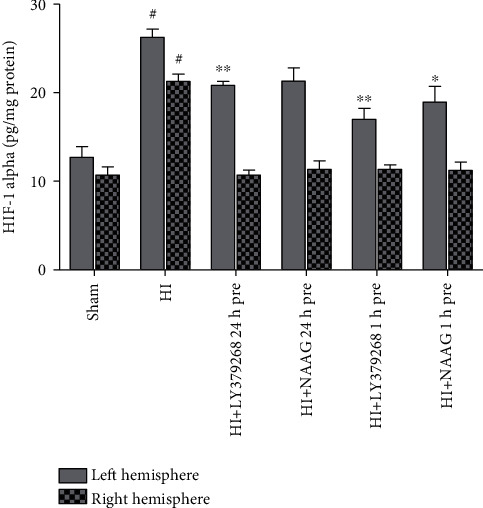
The effect of LY379268 or NAAG application 24 h or 1 h before HI on HIF-1*α* concentration. Results are presented as the mean ± SEM, *n* = 5; statistically significant differences: ^∗^*p* < 0.01 and ^∗∗^*p* < 0.001 compared to HI; ^#^*p* < 0.001 compared to the sham-operated group.

The concentration of HIF-1*α* in brain samples isolated from sham-operated rats was 12.7 pg/mg protein in the left hemisphere and 10.7 pg/mg protein in the right hemisphere. Hypoxia-ischemia significantly increased the concentration of HIF-1*α* to 26.25 pg/mg protein and 21.26 pg/mg protein in the left and right hemispheres, respectively (*p* < 0.001, *F*_1,8_ = 77.82 and *F*_1,8_ = 69.94 for the left and right hemispheres, respectively) (Figure 4).

LY379268 injected 24 h before HI significantly reduced HIF-1*α* concentration to 20.83 pg/mg protein in the left hemisphere (*p* < 0.001), while NAAG application 24 h before HI also reduced HIF-1*α* concentration, though this result was not statistically significant.

Administration of LY379268 and NAAG 1 h before HI significantly decreased the concentration of HIF-1*α* in the left hemisphere to 16.99 and 18.93 pg/mg protein, respectively (*p* < 0.001 and *p* < 0.01 for LY379268 and NAAG, respectively). Application of both agonists decreased HIF-1*α* to control levels in the right hemispheres regardless of the time of administration.

The observed effects of LY379268 and NAAG were comparable and did not differ statistically.

## 4. Discussion

Ischemic tolerance is usually induced by preconditioning with sublethal ischemia or activation of one triggered by ischemia molecular pathways. Preconditioning may have important significance for the development of therapeutic interventions in brain ischemia; however, mechanisms underlying the “ischemic tolerance' phenomenon remain unclear [36]. One of the proposed mechanisms is the activation of glutamatergic receptors both ionotropic and metabotropic. It was shown that activation of mGluR2/3 a short time before or after ischemia or HI attenuates brain injury [27, 37, 38].

Our previous experiments confirmed these observations, showing that application of LY379268 or NAAG not only shortly after HI but also up to 24 h before HI attenuated neurodegeneration [13, 28, 29]. Our results concerning the prolonged effects of pretreatment with LY379268 are in agreement with those presented by Bond et al. [23]. The neuroprotective effect of NAAG applied 1 h before hypoxic exposure but after unilateral common carotid occlusion in a neonatal rat model of hypoxia-ischemia was described by Cai et al. [27]; however, the neuroprotective effect of NAAG applied 24 h before HI was presented for the first time in our laboratory [29]. Moreover, we showed that the neuroprotective effect of NAAG applied 1 h before HI results from a combination of activation of mGluR3 and NMDA receptors, whereas the neuroprotective effect of NAAG applied 24 h before HI is primarily due to NMDA receptors [29]. We also demonstrated that application of both LY379268 and NAAG 24 h or 1 h before HI resulted in a significant reduction in oxidative stress, suggesting that processes leading to apoptotic cell death could also be attenuated [28, 29].

In this study, we investigated the antiapoptotic effects of pretreatment of rat pups with mGluR2/3 agonists before HI. Our results revealed that application of the mGluR2 agonist LY379268 and the specific mGluR3 agonist NAAG up to 24 h before HI results in a decrease in apoptotic activities triggered by the insult. Application of both agonists 24 h or 1 h before HI resulted in a comparable decrease in the number of TUNEL-positive cells in the CA1 area of the hippocampus, indicating inhibition of apoptotic processes. Similar antiapoptotic effects of LY379268 applied a short time before insult were observed in goldfish in a model of the brain and retinal anoxia [39, 40]. The direct antiapoptotic effect of NAAG was observed in glucose-induced cell death [41]. The antiapoptotic effect of increasing NAAG concentration by inhibiting glutamate carboxypeptidase (GCPII), the enzyme that mediates the hydrolysis of NAAG into glutamate and N-acetyl-aspartate, was also previously observed in the ischemia/reperfusion model in adult rats [42].

There are few reports concerning the direct effects of NAAG or LY379268 application on hypoxia-induced increases in proapoptotic factor expression. In our experiments, a significant decrease in HI enhanced Bax, and an increase in Bcl-2 expression was observed after agonist application at both time points before HI. A similar effect was observed in GCPII knockout mice subjected to traumatic brain injury [43] and in the rat model of ischemia/reperfusion when GCPII was inhibited by injection of Id2-siRNA one day before MCAO [42]. The only report on the effect of LY379268 on Bax concentration concerns cultured rat astrocytes subjected to NO exposure. mGluR2 activation by LY379268 prevented astrocyte death by inhibiting Bax activation and decreasing levels of the HIF-1*α* cofactor p53 [44]. The results presented in this study show for the first time the effect of LY379268 application before HI on expression of Bax and Bcl-2 in a rat model of birth asphyxia.

Hypoxia-ischemia significantly increases Bax expression, triggering Bax-dependent mitochondrial outer membrane permeabilization and eliciting the release of cytochrome c. Cytochrome c efflux activates caspase-9, which transforms procaspase-3 leading to its activation [45, 46]. In our experiments, HI-induced increases in caspase-9 and caspase-3 activity were also observed. Application of each of the agonists significantly decreased HI-elevated caspase-9 and caspase-3 activity in the ipsilateral hemisphere, although the effect of LY379268 applied 24 h before HI was more pronounced than the effect of NAAG. The little information available from the literature concerning the effect of LY379268 on caspase activity refers primarily to caspase-3 and is contradictory. Inhibition of caspase-3 activity after mGluR3 activation was observed in the brains of goldfish subjected to anoxia and in cultured astrocytes subjected to oxygen/glucose deprivation [40, 47]. However, Durand et al. [44] did not observe any effect of LY379268 on caspase-3 activity in astrocyte death induced by nitric oxide, concluding that the observed apoptosis was caspase-independent. The lack of an effect of LY379268 applied 5 min after hypoxic exposure on caspase-3 activity was also reported in a neonatal model of HI, and the authors concluded that blockade of caspase-3 activation and the apoptotic pathway is not involved in the neuroprotective effects of LY379268 [24].

The effect of NAAG on HI-increased caspase-9 and caspase-3 is less questionable, as a decrease in the activity of these enzymes was reported in a rat model of focal ischemia [38] and glucose-induced neuronal death [41]. Increasing NAAG levels by inhibiting GCPII activity 1 day before ischemia in rats also resulted in decreased caspase-9 and caspase-3 activity [42]; moreover, an increase in cleaved caspase-3 level in a mouse model of traumatic brain injury was attenuated in the GCPII knockout mice [43].

Our previous results showed that LY379268, as well as NAAG, applied both before and after HI, inhibited the formation of ROS [13, 28, 29]. We presume that the decrease in caspase activity observed in our experiments is derived from the effect of each of the agonists on the development of oxidative stress and prevention of mitochondrial damage.

Increased ROS production contributes to the stabilization of HIF-1*α* and enhancement of its level in cells [48, 49]. Discussion on the exact source of HIF-1*α* stabilizing ROS has been going for several years. Currently, two hypotheses, both connected with mitochondrial dysfunction, are considered: ROS responsible for the increase in HIF-1*α* level after hypoxia/ischemia are either produced by mitochondrial complex III or are dependent on the reduced activity of the mitochondrial electron transport chain as a whole [10]. The results presented in this study demonstrate, for the first time, that application of each of the agonists both 24 h and 1 h before HI significantly decreased elevation by the HI concentration of HIF-1*α*. We suggest that the observed decrease is connected to the reduced ROS production presented in our previous studies [13, 28]. The role of HIF-1*α* in HI-evoked brain damage is also controversial; both neuroprotective and damaging effects of increased HIF-1*α* levels after HI have been described [10, 50]. However, it was shown that accumulation of HIF-1*α* up to 12 h after ischemia correlates with the upregulation of prodeath modulators [51]. In our experiments, the decrease in HIF-1*α* concentration observed after LY379268 or NAAG pretreatment was accompanied by a decrease in apoptotic damage, suggesting that activation of mGluR2/3 receptors before HI resulted in inhibition of HIF-1*α* accumulation during the early phase of apoptosis development.

As we expected, the antiapoptotic effect of each of the agonists was more pronounced when the application was performed 1 h before HI; however, in most analyses, the observed effects did not differ statistically and seemed to be independent of the time of administration.

The mechanism by which LY379268 and NAAG application 1 h before HI inhibits apoptosis most likely results from receptor activation and inhibition/reduction of oxidative stress. However, the neuroprotective effects of NAAG or LY379268 pretreatment 24 h before HI need additional explanation.

It was previously demonstrated that the brain concentration of LY379268 applied in a dose of 10 mg/kg i.p. declines rapidly between 0.5 and 8 h after application, reaching a plateau at a level greater than that required for activation of mGluR2 and remaining at this level up to 24 h [23]. Increased levels of NAAG in the extracellular space, unlike LY379268, are degraded quickly by astrocytic glutamate carboxypeptidase II (GCPII) into glutamate and N-acetyl-aspartate (NAA) [52]. This implies distinct mechanisms of action for each of the agonists applied 24 h before HI.

It was suggested that the prolonged activity of LY379268 after pretreatment 24 or 48 h before ischemia may trigger the same mechanism as that of ischemic tolerance [23]. However, the lack of an additive effect with preconditioning ischemia and the fact that LY379268 pretreatment almost blocked tolerance induced by preconditioning ischemia suggest that neuroprotective effect of LY379268 is connected directly with receptor activation [23].

It has been shown that NAAG, in addition to activating mGluR3, also activates synaptic NMDA receptors containing GluN2A subunits and inhibits extrasynaptic receptors containing GluN2B [53]. The calcium current resulting from the activation of neuronal NMDA receptors by NAAG is comparable to the current induced by NMDA [54] and can trigger the antioxidative defense system, likely through activation of the thioredoxin system [55]. The NAAG degradation catalyzed by GCPII liberates glutamate, which additionally activates extrasynaptic glutamate receptors present on surrounding neurons and astrocytes [56]. We believe that resulting from NAAG application, excessive activation of glutamate receptors may create conditions of mild excitotoxicity, similar to that produced by ischemic preconditioning, triggering the defense mechanisms in neurons. This effect of NAAG pretreatment 24 h before HI fits well with the concept of preconditioning [57].

The present results demonstrate that pretreatment with LY379268 or NAAG applied both 24 h and 1 h before HI reduces the accumulation of proapoptotic factors and as a consequence neuronal death. We connect this effect with the reduction in ROS production that was observed in our previous studies [13, 29], as well as inhibition of ROS-induced apoptotic reactions. While the neuroprotective effect of LY379268 applied 1 h or 24 h before HI seems to be directly connected to the activation of mGluR2, the neuroprotective effect of NAAG applied 1 h before HI results from the combination of mGluR3 and NMDA receptor activation. The effect of NAAG when applied 24 h before HI is likely primarily due to NMDA receptors and may be explained by the induction of defense mechanisms in the manner generally considered to be preconditioning.

## Data Availability

Data can be available on request through the author Elzbieta Salinska—e-mail address: esalinska@imdik.pan.pl.
